# Effect of “Mehrpishegan” web-based support group on depression, anxiety, and stress among elderly informal caregivers: a protocol for a randomized-controlled trial

**DOI:** 10.1186/s13063-022-06351-4

**Published:** 2022-05-17

**Authors:** Fatemeh Rahimi, Elham Shakibazadeh, Mahnaz Ashoorkhani, Hamed Hosseini, Mahshid Foroughan

**Affiliations:** 1 Department of Health Education and Promotion, School of Public Health, Tehran University of Medical Sciences, Tehran, Iran; 2 South Tehran Health Center, Tehran University of Medical Sciences, Tehran, Iran; 3 Center of Research and Training in Skin Disease and Leprosy, Tehran University of Medical Sciences, Tehran, Iran; 4 Clinical Trial Center (CTC), Tehran University of Medical Sciences, Tehran, Iran; 5 Iranian Research Center on Aging, Department of Aging, University of Social Welfare and Rehabilitation Sciences, Tehran, Iran

**Keywords:** Caregivers, Frail elderly, Self-help groups, Mental health, Internet-based intervention

## Abstract

**Background:**

Elderly population in low- and middle-income countries is rapidly growing, which indicates an increase in the number of dependent people needing long-term care. Caring for the elderly is difficult and stressful and threatens physical and mental health of informal caregivers. We aim to design a web-based support group and assess its effectiveness on depression, anxiety, and stress among elderly informal caregivers.

**Methods:**

This is a protocol for a two-arm randomized controlled trial. A total of 160 informal elderly caregivers will be recruited from the southern area of Tehran. Eligible participants will be randomly allocated to two intervention and control groups. The inclusion criteria include not receiving salary for caring, having primary responsibility for care, having smartphone/tablet/computer, being able to use the contents and web applications, having at least one month of experience in caring for the elderly, and having access to the Internet at least once weekly. The intervention will be implemented by giving an account access to the designed website. Depression, anxiety, and stress will be assessed using the DASS21 questionnaire at baseline, and at the end of third and sixth months.

**Discussion:**

Our findings can pave the way for improving the mental health of informal caregivers of the elderly through provision of web-based supportive services. This study stands as an opportunity to address the needs of caregivers and help them support each other in a novel way.

**Trial registration:**

Iran Randomized Clinical Trial Center IRCT20201012048999N1. Registered on 25 December 2020 (current status: ongoing).

The World Health Organization Trial Registration Data Set is in Additional file 1

**Protocol version:**

Second version 2021-05-27

**Supplementary Information:**

The online version contains supplementary material available at 10.1186/s13063-022-06351-4.

## Administrative information

Note: The numbers in curly brackets in this protocol refer to SPIRIT checklist item numbers. The order of the items has been modified to group similar items (see http://www.equator-network.org/reporting-guidelines/spirit-2727-statement-defining-standard-protocol-items-for-clinical-trials/).'

**Table:** 

Title {1}	This protocol describes, single-blind parallel group randomized controlled trial of an online mental health support group system versus no intervention to reduce depression, anxiety, and stress among informal primary caregivers of older adults in Tehran.
Trial registration {2a and 2b}.	This study has been registered in Iran Randomized Clinical Trial Center. Trial Id: 51563 IRCT Id: IRCT20201012048999N1 Registration date: 2020-12-25
Protocol version {3}	V2.0 IRCT Id: IRCT20201012048999N1 Last update: 2021-05-27 Authors: Fatemeh Rahimi Reason for update: Recruitment of participants was postponed and it was decided how to publish the results.
Funding {4}	This study will be conducted as part of a PhD thesis in Health Education and Promotion at Tehran University of Medical Sciences. It is granted by Vice Chancellor for Research, School of Public Health (ID number: 9711108001), and Vice Chancellor for Health at Tehran University of Medical Sciences (Grant number: 1400-1-129-51605).
Author details {5a}	Fatemeh Rahimi^1, 2^, Elham Shakibazadeh^1*^, Mahnaz Ashoorkhani^1^, Hamed Hosseini ^3, 4^, Mahshid Foroughan^5^ 1. Department of Health Education and Promotion, School of Public Health, Tehran University of Medical Sciences, Tehran, Iran 2. South Tehran Health Center, Tehran University of Medical Sciences, Tehran, Iran 3. Center of Research and Training in Skin Disease and Leprosy, Tehran University of Medical Sciences, Tehran, Iran 4 Clinical Trial Center (CTC), Tehran University of Medical Sciences, Tehran, Iran 5. Iranian Research Center on Aging, Department of Aging, University of Social Welfare and Rehabilitation Sciences, Tehran, Iran
Name and contact information for the trial sponsor {5b}	Keshavarz Boulevard, Quds Street, Central University Organization, Vice Chancellor for Research and Technology, Tehran University of Medical Sciences, Tehran, Iran Phone number: +98 81633685- +9881633686 email: vcr@tums.ac.ir
Role of sponsor {5c}	The role of the funders is to keep track of the research planning and execution. Study funders have no role in study design; collection, management, analysis, and interpretation of data; writing of the report; and the decision to submit the report for publication.

## Introduction

### Background and rationale {6a}

Generally, the world’s population is aging. The number of people aged 60 and over, which was about 900 million in 2015, is projected to reach two billion by 2050 [1]. This growth rate varies among countries. It began in high-income countries and is currently increasing in low- and middle-income countries [2]. In Iran, the elderly population is more than seven million people [3], representing 9.2% of the total population in 2016. It is predicted that by 2050, the Iranian elderly population will grow by 10.5% in 2025 and 21.7% in 2050 [4].

Aging of societies will eventually result in an increase in the number of dependent individuals in need of long-term care [5]. Many elderly people lose the ability to live independently due to limited mobility and/or other physical and mental health problems, which makes their needs more pressing for specific kinds of long-term health care and social support. It is estimated that 349 million people worldwide are care-dependent, of which 29% are 60 years old or over. A large number of elderly receive informal care from their family members and/or friends [6].

In recent years, the burden of care is increasing in Iran due to a reduction in household size. Moreover, due to strong emotional ties between family members, and cultural context, families do not place the elderly in long-term care centers [7]. Caring for others is recognized as an important social role that can improve caregiver’s self-esteem and overall well-being; however, it can be a source of stress, tension, unhealthy behaviors, and social restrictions [8, 9]. It may also decrease quality of life of caregiver and increase mortality [8]. Those who care for dependent people find their lives totally affected in a way that they cannot do their own works as well as their family responsibilities [10]. Informal caregiver may not only show care-related stress but also revive conflicts, anger, grief, low self-esteem, frustration, physical burden, time constraints, role pressures, and financial challenges. A long-term family caregiver is sometimes called “a second victim” or “a potential patient” [11].

Caregivers for disabled individuals are at risk of developing depression. In a study conducted in China, it was reported that the prevalence of depression among caregivers was 37.7% [9]. Depression adversely influences behavior, cognition, and emotions and significantly reduces the quality of care. Moreover, depression may lead to physical pain and even raise the tendency to commit suicide [11].

Anxiety is another important negative emotional implication of care, for which the rate depends on the type of care. The anxiety rate ranges from 21.4% among caregivers of stroke patients to 43.6% in caregivers of dementia patients [10].

Informal caregivers rely on a wide range of internal and external resources for health information, support, and counseling, including health-care professionals, printed materials, and web-based resources, to do their job properly. Among support group interventions, the ones that provide interactions with peers and professionals, e.g., web-based support groups, are of particular importance [12]. Caregivers can use support group websites to search for information, receive emotional support, use the experience and advice from other caregivers, and learn how to better self-care [12, 13]. Studies have also highlighted the positive effects of web-based support groups, including reducing loneliness, gaining identity, giving a sense of social inclusion, belonging, comfort and acceptance, hope, and a safe environment [12–14]. These web-based support groups are deemed a feasible and cost-effective intervention to support caregivers. Instant and easy access to these groups allows members to avoid the need to leave the care recipient, thus removing a main barrier to participating, i.e., spending time to attend face-to-face meetings and the need to find an alternative caregiver [13]. In addition, in-person support groups may be difficult to access due to high health care workload and/or geographical restrictions [14]; multiple daily activities such as housekeeping, employment, and caring for other family members; and fear of being labeled [15].

In Iran, a number of support groups and self-help interventions have been implemented and have resulted in fruitful results. However, no study is available to assess the effect of web-based support groups among elderly caregivers. This protocol aims at proposing the backbone for a study with the purpose of assessing the effect of a web-based support group entitled “Mehrpishegan” on depression, anxiety, and stress in informal primary caregivers of elderly in Tehran.

## Objectives {7}

This is the protocol for our study, which aims to assess the effect of Mehrpishegan web-based support groups on depression, anxiety, and stress in elderly informal primary caregivers. The hypothesis of this study is that there is a difference in the status of depression, anxiety, and stress among the participant caregivers before and after implementing the intervention.

## Trial design {8}

This is a parallel group, two-armed, superiority, single-blinded, randomized trial. The number of participants in intervention and control groups will be equal. Randomization will be performed using block randomization with a 1:1 allocation. The control group will not be exposed to the intervention during the study.

## Methods: participants, interventions, and outcomes

### Study setting {9}

This study will be conducted in the South Tehran, which includes four urban and two rural areas. The healthcare services in the South Tehran are covered by the South Tehran Health Center. The population coverage of the whole area is more than three million people; of those, about 80,000 are over 60 years old [16]. We will be in contact with 15 local health centers and two hospitals from this area that provide health and medical care to the elderly in order to recruit the informal caregivers (Additional file 2).

### Eligibility criteria {10}

The inclusion criteria include not receiving salary for caring, having primary responsibility for care, having smartphone/tablet/computer, being able to use the contents and web applications, having at least 1 month of experience in caring for the elderly, and having access to the Internet at least once weekly. Any informal caregiver diagnosed with mental illness and undergone treatment, those participating in other support groups (virtual or non-virtual), or non-Iranian caregivers will be excluded.

### Who will take informed consent? {26a}

F.R. will introduce the study and explain how to confirm the consent form via telephone. In addition, participants will be instructed to obtain information about the study from the “research information” and “about us” pages on Mehrpishgan website. Then, participants will complete and confirm the electronic consent form (Additional file 3) uploaded on Mehrpishgan website.

### Additional consent provisions for collection and use of participants’ data and biological specimens {26b}

This is not applicable. No biological specimens will be collected for research purposes, so additional consent from the participants will be not required.

### Interventions

#### Explanation for the choice of comparators {6b}

All recruited participants will be randomly allocated to one of the intervention or control groups after baseline measurement. The intervention will not be applied to the control group during the study. They will receive routine care during the trial. If the intervention would be effective, at the end of the study, the control group participants can use the contents of the website and take advantage of the support group in order to ethical consideration.

#### Intervention description {11a}

To form a web-based support group, we will provide a virtual, interactive, secure, and confidential information website in Persian language. To prepare the format and content of this website, we will assess the informational needs of the informal caregivers of elderly using a qualitative study. Then, we will review the existing printed and electronic materials and consult experts in the field. In addition, we will tap into similar websites, software, and applications.

We will use a content management system (WordPress). This website is compatible with a variety of devices, including smartphones, tablets, and PCs (responsive websites), so that people with different devices can use it. We prefer developing the website to mobile applications because it does not require software installation and will not take up a large space on electronic devices. In addition, websites are easily accessible using URLs and do not require specialized knowledge or particular computer skills to use. It also will be employable with public internet's standard speed. Furthermore, we will define different access levels based on the user type (intervention group, control group, and research team).

To achieve the study objectives, we will prepare a form to record the demographic characteristics of participants, an online questionnaire, an online chatroom, two asynchronous forums, an educational section, a form to send feedback questions and suggestions to the website manager (PI), and research guides and information pages to introduce research team members, describe the study objectives and the advantages and disadvantages of participation, and provide instructions for using the website.

After designing, Mehrpishegan website will be tested by a small group of users and then will be revised and fixed accordingly.

#### Intervention procedure

##### The Web-based support group

The intervention will be in the form of using Mehrpishegan website. The content of Mehrpishegan will include the skills needed by caregivers to care for the elderly and to manage their own mental health.

During the first 3 months, interactions between intervention group members in the online chatroom will take place through two theoretical and practical parts. In the theoretical part, information on the topic will be provided once a week at a certain time by the facilitator (a psychologist), and the intervention group will be encouraged to share information, problems, concerns, feelings, experiences, life stories, and possible solutions with their teammates and experts. The average duration of each session in an online discussion will be from 60 to 90 min. In the practical part, the facilitator will provide practical assignments to the members of the intervention group. Furthermore, members in the intervention group will be guided to use asynchronous forums to discuss questions and answers related to the elderly care and their own mental health issues. The participants can discuss their assignments in the chatroom and ask questions, as well as submit questions about uploaded educational content and receive answers accordingly. In the second 3 months, active members of the support group will lead online group discussions without the direct involvement of professional team members.

##### The control group

They will have access to the general interface of the website; but they cannot use facilities related to the intervention.

#### Criteria for discontinuing or modifying allocated interventions {11b}

Termination of care tasks (change of care status to non-primary caregiver, death or recovery of the elderly); the participant’s request; leaving the support group and not using the website; undergoing any kinds of medical treatments; emergence of an adverse issue during the study that seriously affects the individual’s mental health, such as divorce or death of close relatives; or any behavior that threatens or harms other participants will result in exclusion from the study or discontinuing the intervention.

To estimate the efficacy, all participants will be analyzed according to randomization assignment (ITT approach) as a full analysis set (FAS) population. In addition, per protocol (PP) approach will be done as well.

#### Strategies to improve adherence to interventions {11c}

Logging in to the website will be through a personal username and password, and approval of the administrator (FR) in order to maintain confidentiality and protect adherence. Participants in the interventional group will learn how to properly use different parts of the website such as support group, forums, and educational content. In addition, participants will be reminded to join the group and forums at the appropriate times via short message service (SMS)/notifications. Participants will be encouraged to take part in discussions and read the summary file prepared by the facilitator. Also, the researchers will participate in the support group and forums in order to increase their common sense and oversight. Participants will be able to send their comments and suggestions through the website and contact the researcher/facilitator in case of any problems.

#### Relevant concomitant care permitted or prohibited during the trial {11d}

Since the informal caregivers for elderly are not registered as caregivers in any health or social care system, they do not receive any special care services. As a result, participants will be able to obtain the same health and social services as the general public during the trial, but they will be excluded if they require mental health therapy.

#### Provisions for post-trial care {30}

The website will be maintained to answer the questions of the participants about elderly care post-trial. Participants can use a free consultation with a psychologist, attend a psychology workshop, or receive free related books after the trial. Although the nature of the study is educational and supportive and does not appear to be harmful, in the event of reporting adverse effects, researchers will be responsible for following up until the problem is resolved or paid for.

### Outcomes {12}

This study has three primary outcome variables (depression, anxiety, and stress) that are going to be measured by the DASS-21 scale three times during the study: before (T0), by the end of the first 3 months of the intervention (T1), and by the end of the second 3 months (T2) after the intervention. The effect of the web-based support group on depression, anxiety, and stress in the intervention and control groups will be assessed and compared before and after the intervention. The difference in the mean scores of the outcomes will be assessed.

### Participant timeline {13}

This study’s participant recruitment began in August 2021 and is still ongoing. The recruitment process is expected to be completed in March 2022. The intervention, data analysis, final report, and knowledge translation activities will take place between March 2022 and December 2023. This manuscript was designed according to SPIRIT reporting guidelines [17].

Time schedule of enrolment, intervention, and assessments is illustrated in Table 1 according to the SPIRIT Guideline.

**Table 1:** Participant’s timeline in Mehrpishegan study

	Study period				
	Enrolment	Allocation	Post-allocation		Close-out
Timepoint	−t1	t0	t1	t2	t3
**Enrolment:**					
Eligibility screen	X				
Informed consent	X				
Learning to use the website and assign a username and password	X				
Allocation and randomization			X		
**Interventions:**					
**Intervention** (first 3 months)			X		
**Intervention** (second 3 months)				X	
**Assessments:**					
Baseline sassement: Demographic questionnaire + Dass-21		X			
Assessment (T1 Dass-21				X	
Assessment (T2): Dass-21					X

### Sample size {14}

The study population consists of informal primary caregivers of the elderly in the South Tehran, who have the willingness to participate. The sample size has been calculated using PASS 15.0.5 software. Based on literature [18] and using Cohen’s formula (expecting the average effect size) with a coefficient = 0.5 φ [19], for both of the intervention and control groups (63 people), taking into account the power of 80. The effect size is 0.50 and the significance level (alpha) will be 5%, the probability of drop-out and withdrawal of the study is 20%, equivalent to 80 people in each group, and the total will be 160 people.

### Recruitment {15}

We will seek help from the South Tehran Health Center, hospitals, and other organizations in the area to cooperate to promote adequate enrolment. We will explain the aims and process of the study to their staff. We will ask them to advertise our study on virtual social networks and invite caregivers to participate in it. We will continue advertising until we reach the expected sample size. Furthermore, we will review the health records of elderly people in order to identify dependent elderly people and their caregivers, which were compiled in the South Tehran Health Center. Informal caregivers will be invited to participate according to inclusion criteria using telephone invitations. The purpose of the study, possible pros and cons of participation in the study, assurances of confidentiality, and the process of conducting the study will be explained to the caregivers. Also, the Mehrpishgan website will be introduced. If the individuals are interested in participating in the study and are eligible, they will receive a username and password and will be asked to complete the online consent form and the baseline measurement on the website. Before the commencement of the intervention, participants who will be in mild and moderate categories (based on DASS-21 scores) will be randomly assigned to one of the intervention or control groups.

Afterwards, participants in the intervention group will be asked to establish their activities in the web-support group for 6 months. At the end of the third month and sixth month, T1 and T2 assessments will be conducted in both intervention and control groups.

**Fig. 1:**
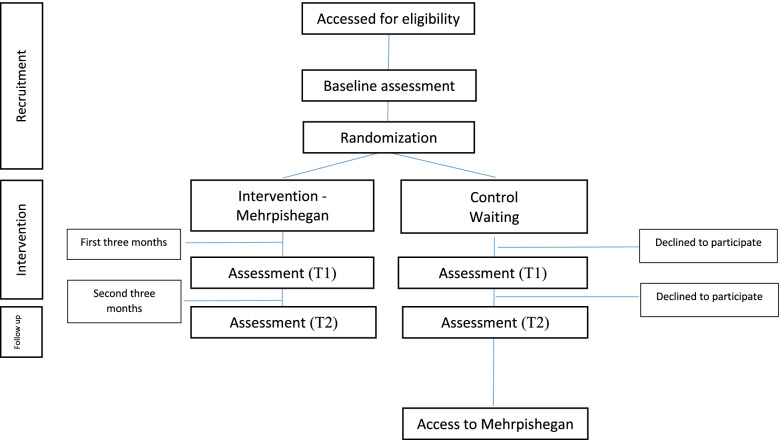
Participations flow diagram

The control group will not be exposed to any intervention during the study. However, after completing the T2, they can use facilities of the website (Fig. 1).

## Assignment of interventions: allocation

### Sequence generation {16a}

Randomization sequences will be generated using the online sealed envelope website (https://www.sealedenvelope.com/). To generate this randomized sequence, firstly a Software of Statistics and Data Sciences statistical (STATA) will be generated on sex variable; then, based on block size of four, all individuals will be allocated to two equal groups (20 blocks). A statistical consultant, independent of the research team, will generate a random sequence.

### Concealment mechanism {16b}

For each person in each group, anonymous codes will be generated by employing the concealment code. Instead of the sequence of intervention groups as A and B, the concealment codes will be provided to the researchers in the field, and the randomization sequence will be blind during random assignment.

### Implementation {16c}

Potential participants will be invited to the study by the staff of the above-mentioned centers. HH will use sealed envelope website to produces the allocation sequence. The study groups will be revealed only to the FR.

## Assignment of interventions: blinding

### Who will be blinded {17a}

This will be a single-blind study. Primary caregivers will be invited to the study through the researcher’s phone call. Then, invited caregivers who meet inclusion criteria will register on the website and fill in the baseline measurement (DASS-21). The score will be calculated by the website plugin automatically at the end of the questionnaire. Based on the scores, participants in the mild and moderate categories will be informed that they will be randomly divided into two groups: the intervention group, who will have access to the website’s support group for six months, and the control group, who will only have access to the general interface of the website during the study. The data will be entered to SPSS software and delivered in coded form (first and second group) to data analyst to avoid bias in the outcome evaluation, so only the data analyst will be blinded.

### Procedure for unblinding if needed {17b}

This is not applicable.

## Data collection and management

### Plans for assessment and collection of outcomes {18a}

Data will be collected in three stages: before the intervention (T0), by the end of the first 3 months (T1), and by the end of the second 3 months (T2) after the intervention, using a 22-item demographic questionnaire and the online standard Persian-version of the 21-item DASS questionnaire.

The demographic questionnaire consists of 22-items including four main parts: (1) demographic characteristics of the caregivers including age, gender, educational status, occupation, marital status, place of residency, health status, and ethnicity; (2) activities of the caregivers including passing an elderly care training course, using comprehensive health care services, social activities, and types of services received for care assistance; (3) records on how to provide care, such as duration of care, daily life activities, instrumental daily life activities, number of people receiving care, and availability of the needed care; and (4) demographic characteristics of the elderly, including age, gender, health status, insurance, and ethnicity.

The DASS-21 Standard Depression, Anxiety, and Stress Questionnaire consists of 21 items prepared by Lovibond and Lovibond in 1995. The validity and reliability of the Persian-version of the questionnaire have been checked among Iranian population in some previous studies [20–22].The questionnaire is designed on a 4-point Likert scale: zero = at all (never), 1= low (sometimes), 2 = medium (often), and 3 = high (almost always) options. The lowest score for each question is zero, which indicates the best situation, while the highest score is three, which indicates the worst condition. To calculate the final score on each scale, the set of scores is multiplied by two, and the participant is placed in one of the normal, mild, moderate, severe, very severe subgroups specific to that scale. The points are calculated by the website plugin and are provided to the participant automatically at the end of the test.

### Plans to promote participant retention and complete follow-up {18b}

Study participants will be invited to the study by telephone and will receive full information about the study, how to use the website, and the importance of completing it. Questionnaires are uploaded on the website so that each participant can answer the questions accurately without the stress of time constraints. At each stage, participants are reminded via SMS to complete the questionnaires. During the study period, researchers review the answers and contact participants, if necessary. Researchers will also be available to answer potential questions via the website or by phone. Intervention team members will be notified and encouraged to join the online chatroom and use site updates related to the intervention via SMS.

### Data management {19}

All information, including informed consent forms and demographic characteristics, will be collected through Mehrpishegan website in the form of electronic case report forms (eCRF). The questionnaires are also completed online by the participants. The database will be backed up every day. Total backups will be done weekly and monthly. A password system will be utilized to control access to the study data.

### Confidentiality {27}

Each participant in the intervention group will have a unique username and password in order to use the website, so, their privacy will not be violated in this regard. Research data is stored using a study identification code for each participant. The key to the identification code list will only be available to the research team during the study and will be documented and protected by FR after completion of the study. No participant identification information will be reported in the related publications. The technical support company for the website will be committed to maintaining the confidentiality of the users' information.

### Plans for collection, laboratory evaluation, and storage of biological specimens for genetic or molecular analysis in this trial/future use {33}

This is not applicable. This study has no laboratory evaluation or storage of biological specimens for genetic or molecular analysis, according to the researchers.

## Statistical methods

### Statistical methods for primary and secondary outcomes {20a}

All eligible participants in the study who will be randomly assigned to the groups will be considered the Full Analysis Set (FAS) that will make up the intent to treat analysis (ITT) population. The per-protocol population is a subset of the ITT population that has no violations of the study protocol (type of intervention and timing specified). Information on all quantitative data will be reported as mean (standard deviation) or median interquartile range (IQR), while qualitative data will be reported as frequency and percentage. We will compare the two groups to measure the effectiveness of the intervention and changes in the outcomes (depression, anxiety, and stress) over time using the repeated measures ANOVA test. The SPSS and STATA will be used for analysis of data.

### Interim analyses {21b}

This is not applicable. There are no interim analyses planned.

### Methods for additional analyses (e.g., subgroup analyses) {20b}

This is not applicable. There are no subgroup analyses planned.

### Methods in analysis to handle protocol non-adherence and any statistical methods to handle missing data {20c}

We will use the intention to treat (ITT) analysis approach. For missing data, simple linear regression methods will be used to replace the missing data (imputation).

### Plans to give access to the full protocol, participant-level data, and statistical code {31c}

The corresponding author can provide the full protocol information and the relevant data analyzed during the development of this study upon request.

## Oversight and monitoring

A team from Vice Chancellor for Research at Tehran University of Medical Sciences will monitor the study’s implementation and datasets and will potentially make recommendations for protocol changes or the termination of the study.

### Composition of the coordinating center and trial steering committee {5d}

This study is part of a PhD thesis in Health Education and Promotion program, so the main responsibility for preparing the protocol, and implementation, including identification, recruitment, data collection, and implementation of the intervention, along with following the study participants and publishing study reports, is the responsibility of the student (F.R.).

In addition to the student, the members of the research team include the supervisors and advisors (two professors in Health Education and Promotion, one epidemiologist with a specialty in clinical trial projects, and one psychiatrist). The project supervisor and advisors are responsible for reviewing the protocol, monitoring the proper implementation of the project from a scientific and executive point of view. They are also responsible for the progress of the study, agreeing to change the protocol if necessary, and confirming the accuracy of the results for publication.

Budget management is done jointly by E.Sh and F.R. E.Sh will provide the research findings to the Vice Chancellor for Research and the Vice Chancellor for Health at Tehran University of Medical Sciences. In the event of any danger or unwanted damages, E.Sh and F.R. are responsible for reporting it to the ethics committee.

Furthermore, the project will benefit from the cooperation of experts in software for technical support of Mehrpishegan website, psychologists, geriatricians, and nutritionists to provide educational content, facilitate the support group, and answer questions from participants.

The main research team members will meet at the end of each trial phase and whenever needed. The other members will be in touch throughout the research and will meet as needed at each step.

### Composition of the data monitoring committee, its role, and reporting structure {21a}

The Vice Chancellor for Research at Tehran University of Medical Sciences will be in charge of data monitoring. The auditors will follow a monitoring plan to ensure that the clinical trial is carried out and that data is generated, documented, and reported in accordance with the protocol and regulatory requirements.

### Adverse event reporting and harms {22}

Due to the nature of the educational and supportive interventions, the health of the participants in this study will not be at risk, and no harm is expected.

We stated in the consent form that if any unexpected harm occurs to the participants during and after the study due to their participation in the study, the treatment of complications and its costs will be the responsibility of the research team members.

During the study, all adverse events (related or unrelated to the intervention) will be collected by the follow-up team according to the timelines. For all collected adverse events, causality assessment will be performed. All events will be reported individually. Adverse events will be reported based on MedDRA dictionary.

### Frequency and plans for auditing trial conduct {23}

There will be unplanned checks on the quality of the data or the progress of the trial. Auditing is considered by exploring the trial dataset and arranging site visits if necessary. Auditors are independent from the trial investigators.

### Plans for communicating important protocol amendments to relevant parties (e.g., trial participants, ethical committees) {25}

Any major changes in the project implementation method that may affect the potential benefit, safety, and physical or mental health of the participants will be agreed upon by the authors and will be notified to the Ethics Committee and related organizations prior to implementation. In addition to registration, changes will be mentioned to the participants, and supplementary consent will be added.

## Dissemination plans {31a}

We plan to present the findings of this study to key stakeholders at related seminars and publish them in peer-reviewed journals. The dataset is available upon request from the corresponding author. In addition, a copy of the thesis will be provided to the South Tehran Health Center. Upon request of the participants, a plain summary of the results will be announced to them.

## Discussion

The results of previous studies on caregivers’ mental health have shown some beneficial effects of Internet-based interventions in reducing symptoms of depression, stress, and anxiety. Based on these studies, it seems that the type of Internet-based intervention (providing information and training alone, providing information and training plus psychological support of professionals, peers, or both) affects the results [18].

In Iran, the number of interventional studies conducted on the effects of support and self-help groups in maintaining and improving the mental health status of caregivers is small [23–27], even though previous studies have shown that Iranian caregivers could benefit from participating in these groups. Assessing the effects of an educational support group on the distress of family caregivers of dementia patients [23], implementing emotional support sessions to reduce stress and improve spiritual health of family caregivers of elderly people with Alzheimer's disease [24], conducting peer support groups on the mental well-being of spouses of veterans with post-traumatic stress disorder [25], training peers to reduce the mental burden of family caregivers of schizophrenia patients admitted to military hospitals [27], and training caregivers of dementia patients [28] are among these studies. However, no study is available to assess the effects of web-based support groups on improving the mental health status of this high-risk group.

There is also no comprehensive national system in Iran to record the information of informal caregivers in order to support, plan, analyze, and meet the health needs of this group of people. These days, the services provided to the caregivers are scattered; hence, there is a sort of discoordination. Moreover, most services provided are face-to-face and not web-based. Therefore, they can just offer limited, intangible, and disrupted help that are only provided to a few numbers of caregivers. Also, the number of current support groups for informal caregivers of the elderly is insufficient to cover the targeted population. In addition, many caregivers cannot leave the elderly to attend face-to-face support groups, especially in the COVID-19 pandemic.

Mehrpishegan is going to be a virtual website that has simultaneous and asynchronous chatrooms and offers advice to informal caregivers among older people on maintaining mental health and preventing mental disorders. Furthermore, it provides educational materials on common problems of the elderly and ways to handle such issues. In this study, we will focus on empowering people through mutual help and reciprocal assistance. This study can help in maintaining the mental health of informal caregivers of the elderly and preventing the development of mental health disorders caused by stressful caring conditions. Our goal in this study is to support caregivers when sharing life problems, worries, fears, feelings, and experiences. We also aim to empower caregivers to seek credible information sources and provide mutual aid as a social responsibility through a web-based support team. We seek to maintain and promote the mental health of caregivers by reducing their sense of loneliness and enabling them to find social identity through interaction with others.

This study will provide an opportunity to improve the caregivers’ mental health to better cope with their situation. We hope this study can pave the way to developing proper national policies and provide a more comprehensive model tailored to the resources, infrastructure, and cultural context.

This study can be a prelude to planning the activities of the Ministry of Health and Medical Education, and the health system, governmental and non-governmental organizations, to provide web-based services to informal caregivers of the elderly, and guide other researchers to design and conduct similar web-based interventions that are more accessible in present conditions.

This study faces some limitations, including the difficulty of forming different types of support groups and the inability of some groups of caregivers, such as the aged and illiterate, to work with these proposed websites, along with other limitations such as lack of time that may reduce the number of potential caregiver participants or even increase the rate of attrition.

## Trial status

This study has been recorded as a trial in Iran Randomized Clinical Trial Center (IRCT20201012048999N1 on December 25, 2020). Recruitment started in August 2021 and is still continuing. The recruitment process is expected to be completed in March 2022.

Current protocol version and date: V2.0, Last update: 2021-05-27.

## Supplementary Information

**Additional file 1.** WHO data set

**Additional file 2.** The list of centers

**Additional file 3.** English version consent form

## Data Availability

To protect confidentiality, individual participants’ data cannot be shared publicly. However, the dataset used or analyzed during the current study will be available for researchers upon request from the corresponding author.

## Abbreviations

DASS21: Depression, Anxiety, and Stress Scale
CMS: Content Management System
SMS: Short message service
eCRF: Electronic case report form
SPSS: Statistical Product and Service Solutions
STATA: Statistics and Data Sciences statistical
IQR: Interquartile range
ITT: Intention to treat
PI: Principal investigator
