# Supplementary material for: Effect of “Mehrpishegan” web-based support group on depression, anxiety, and stress among elderly informal caregivers: a protocol for a randomized-controlled trial
Source: Trials. 2022 May 17;23:413. doi: 10.1186/s13063-022-06351-4 (PMC9110945; doi:10.1186/s13063-022-06351-4)
Supplement: Supplementary file 1 — Additional file 1. WHO data set [file 13063_2022_6351_MOESM1_ESM.pdf]

WHO Trial Registration Data Set

	Data category	information
**1**	**Primary Registry and Trial Identifying Number**	IRCT Id: IRCT20201012048999N1
**2**	**Date of Registration in Primary Registry**	2020-12-25
**3**	**Secondary Identifying Numbers**	IRCT Id: IRCT20201012048999N1
**4**	**Source(s) of Monetary or Material Support**	Tehran University of Medical Sciences
**5**	**Primary Sponsor**	Vice Chancellor for Research of the School of Public Health, Tehran University of Medical Sciences
**6**	**Secondary Sponsor(s)**	Vice Chancellor for Health of Tehran University of Medical Sciences
**7**	**Contact for Public Queries**	• **Full name of responsible person**: Fatemeh Rahimi<br>• **Position**: PhD student<br>• **Latest degree**: Master<br>• **Other areas of specialty/work**: Health Promotion<br>• **Street address**: Department of health education and promotion,School of Public Health, Tehran University of Medical Sciences, Poursina Avenue, Qods Street, Enqelab Square, Tehran, Iran.<br>• **Postal code**:1417613151<br>• **Phone**:+98 21 8898 9128<br>• **Mobile**:+98 9386545847<br>• **Email:** f-rahimi@razi.tums.ac.ir
**8**	**Contact for Scientific Queries**	• **Full name of responsible person**: Fatemeh Rahimi<br>• **Position**: PhD student<br>• **Latest degree**: Master<br>• **Other areas of specialty/work**: Health Promotion<br>• **Street address**: Department of health education and promotion ,School of Public Health, Tehran University of Medical Sciences, Poursina Avenue, Qods Street, Enqelab Square, Tehran, Iran.<br>• **Postal code**:1417613151<br>• **Phone**:+98 21 8898 9128<br>• **Email:** f-rahimi@razi.tums.ac.ir
**9**	**Public Title**	The effect of internet support group on the mental health of the elderly family-caregivers
**10**	**Scientific Title**:	Effect of web support groups on depression, anxiety and stress among informal primary caregivers of the elderly.
**11**	**Countries of Recruitment**	Iran
**12**	**Health Condition(s) or Problem(s) Studied**:	Participants are volunteers who are classified as mild and moderate in depression, anxiety and stress according to the DADD21 questionnaire
**13**	**Intervention(s)**	WEB SUPPORT GROUP: Participants in the intervention group will participate in a web support group for 6 months. During the first 3 months, the interactive content of group consists of two parts:In theoretical part information about a topic will be provided by the facilitator.The members will share their concerns, feelings and experiences, life stories and possible solutions with their peers accordingly. The facilitator will provide practical tasks for members during the time assigned for the practical part.The members can also talk about homework and receive feedback from the facilitator and other peers. Members will be guided to receive more content in other parts of the website. Two

		participants will lead the support group without the guidance of the professional team during the next 3 months.<br><br>CONTROL GROUP: It will not receive any intervention during the study. In order to ethics, the members of the control group can use the contents of the website and participate in the support group after the end of the intervention and collecting the third stage questionnaires.
**14**	**Key Inclusion and Exclusion Criteria**	• Iranian Informal primary caregivers of elderly whose age is at least 18 years old in the southern Tehran can participate in the study<br>• **Minimum age:** 18 year<br>• **Maximum age:** not age limit<br>• **Gender:** Both<br>• **Inclusion criteria**<br>Provision of non-paid care Having the primary responsibility for care Having a smart phone / tablet / computer Ability to use web content and applications Caring of the elderly at least 6 hours a week Having an experience of at least 1 month of elderly care Ability to communicate in Persian Education level not less than 9 grades Access to internet at least once a week<br><br>• **Exclusion criteria**<br>• Psychiatric disorders under treatment based on persons' statements Participate in other support groups (virtual or non-virtual) non-Iranian Caregiver or elderly
**15**	**Study Type**	• Type of study: interventional<br>• Study design including:<br>• Method of allocation: (randomized)<br>Masking: Participants will be informed that they will be randomly divided into two groups after completing the first questionnaire(T0): the first group of people who will have access to the support group for six months immediately after completing the first questionnaire(T0) and the second group of people who will have access to the support group after completing the third questionnaire(T2).<br>The collected data is delivered in coded form (first and second groups) to the data management team. To avoid bias in the outcome evaluation, research assistants who are responsible for analysis will be blinded to the participant allocation.<br>• Assignment: parallel<br>• Purpose: to reduce depression, anxiety, and stress among informal primary care-givers of older adults<br>Sequence generation: Randomization sequences were generated using the online sealed envelope website (https://www.sealedenvelope.com/). To generate this randomized sequence, first a class was set based on gender and then based on block size of four, all individuals were allocated to two equal groups (20 blocks).<br>A statistical consultant independent of the research team generated a random sequence.<br>Concealment mechanism: For each person in each group, anonymous codes will be generated by employing the concealment code. Instead of the sequence of intervention groups as A, B, the concealment codes will be provided to the researchers in the field, the randomization sequence will be blind during random assignment.

**16**	**Date of First Enrollment**	2021-08-03
**17**	**Sample Size**	160
**18**	**Recruitment Status**	Recruiting
**19**	**Primary Outcome(s)**	• Outcome Names: Depression, Anxiety, Stress<br>• Metric/method of measurement: Depression, Anxiety, Stress Scale 21 items questionnaire<br>• Timepoint: Measurement of depression at the beginning of the study (before the intervention) and 3 months and 6 months after the baseline
**20**	**Key Secondary Outcomes**	-
**21**	**Ethics Review**	• Status: Approved<br>• Date of approval: 2020-12-02<br>• Name and contact details of Ethics committee(s): Ethics committee of School of public health and allied medical sciences-Tehran University of Medical<br>• Address: School of Public Health, Tehran University of Medical Sciences, Poursina Avenue, Qods Street, Enqelab Square, Tehran, Iran. Postal code : 1417613151<br>• Ethics committee reference number: IR.TUMS.SPH.REC.1399.226
