# Supplementary material for: Effect of “Mehrpishegan” web-based support group on depression, anxiety, and stress among elderly informal caregivers: a protocol for a randomized-controlled trial
Source: Trials. 2022 May 17;23:413. doi: 10.1186/s13063-022-06351-4 (PMC9110945; doi:10.1186/s13063-022-06351-4)
Supplement: Supplementary file 2 — Additional file 2. The list of centers [file 13063_2022_6351_MOESM2_ESM.pdf]

Additional file 2

The list of centers will be involved in trial (Effect of Mehrpishegan" web-based support group in reducing depression, anxiety and Stress among the elderly informal caregivers: A protocol for a randomized-controlled trial)

1- Shoja fard local health center, South Tehran Health Center, TUMS, Tehran, Iran (17 urban area, Imamzade Hasan St).
2- Shahid Ayad local health center, South Tehran Health Center, TUMS, Tehran, Iran (17 urban area, Mahan St).
3- Meisam local health center, South Tehran Health Center, TUMS, Tehran, Iran (17 urban area, Ghazvin St).
4- Aboozar local health center, South Tehran Health Center, TUMS, Tehran, Iran (17 urban area, Aboozar St).
5- Farmanfarmaian local health center, South Tehran Health Center, TUMS, Tehran, Iran (11 urban area, EaSt Azarbaijan St).
6- Salas local health center, South Tehran Health Center, TUMS, Tehran, Iran (11 urban area, Vahdat Islami St).
7- Ebne sina local health center, South Tehran Health Center, TUMS, Tehran, Iran (11 urban area, Navab safavi St).
8- Akbar abad local health center, South Tehran Health Center, TUMS, Tehran, Iran (10urban area, Navab safavi).
9- 10-No local health center, South Tehran Health Center, TUMS, Tehran, Iran (10urban area, Khosh St).
10- Ghamar bani hashem local health center, South Tehran Health Center, TUMS, Tehran, Iran (10urban area,Jey St).
11- 14 Masoom local health center, South Tehran Health Center, TUMS, Tehran, Iran (10urban area, Ghazvin St).
12- Shahi Vahedi local health center, South Tehran Health Center, TUMS, Tehran, Iran (16urban area, Yakhchi abad St).
13- Bakhsh e nooshesh local health center, South Tehran Health Center, TUMS, Tehran, Iran (10urban area, Nazi Abat St).
14- Imam Mohamadbagher local health center, South Tehran Health Center, TUMS, Tehran, Iran (19 rural area, Mortezagerd Town).
15- Imam sadegh local health center, South Tehran Health Center, TUMS, Tehran, Iran (19 rural area, Ahmadiye Town).
16- Imam Khomeini hospital complex, TUMS, Tehran, Iran
17- Ziaeian hospital, TUMS, Tehran, Iran.
