# Supplementary material for: Effect of “Mehrpishegan” web-based support group on depression, anxiety, and stress among elderly informal caregivers: a protocol for a randomized-controlled trial
Source: Trials. 2022 May 17;23:413. doi: 10.1186/s13063-022-06351-4 (PMC9110945; doi:10.1186/s13063-022-06351-4)
Supplement: Supplementary file 3 — Additional file 3. English version consent form [file 13063_2022_6351_MOESM3_ESM.pdf]

Consent Form

Dear participant,

You are hereby invited to participate in a study titled "**The effect of web support groups on depression, anxiety, and stress in informal primary caregivers of the elderly in the south of Tehran: a mixed study (qualitative and clinical trial)".**

You are free to participate or not participate in this research. You do not have to make an immediate decision. To decide on this, you can ask your questions to the research team and consult anyone you want. Before signing this consent form, make sure that you have understood all the information in this form and all your questions have been answered, because pressing the start option means your consent to participate in this research. Principal Researcher: Fatemeh Rahimi

1.I know that the purpose of this study is to investigate the effect of participating in web-based support groups on the rate of depression, anxiety, and stress in nursing home caregivers.

2.I know that my participation in this research is completely voluntary and that I do not have to participate in this research.

3.I was assured that if I refused to participate in this study, I would not be deprived of routine health, diagnostic, and treatment care, and my relationship with the healthcare team would not be compromised.

4.I know that even after agreeing to participate in the research, I can leave the research whenever I want, after informing the facilitator, and my withdrawal from the research will not deprive me of receiving the usual services of organizations for me.

5.How can my participation in this research be tailored to the needs of the research team as follows:

- Completing a personal profile questionnaire for myself and the elderly
- Complete a questionnaire of 21 questions, at least three times.
- Using the facilities of the site, such as participating in Internet support groups, using educational materials, and doing relevant homework
- Participate in a personal interview (IF NECESSARY).

6: The use of educational materials and support provided by peer groups and experts in person or online are potential benefits of my participation in this study. I can attend a free counseling session or workshop or get a free research book at the end of the study.

7-I know that participating in this study has no harm or side effects for me. I just have to spend my time completing questionnaires and participating in discussions according to the rules.

8-I know that if I do not want to participate in the study, I will not be in any danger and I will not be harmed. Not wanting to participate in this study will not prevent me from receiving care services normally. It will also not be a problem for me to use virtual networks.

9-I know that if during and after the research any problems, both physical and mental, occur to me due to participating in this research, the treatment of complications, its costs, and related compensation will be the responsibility of the executor.

10.I know that I will not be responsible for any of the costs of this study, including completing questionnaires and participating in discussion groups.

11.I know that those involved in this research keep all information about me confidential and are only allowed to store my answers and only the general and group results of this research without mentioning my name and details.

12.Ms. Fatemeh Rahimi was introduced to me to answer and I was told to share any problem or question regarding participating in the mentioned research with her and ask for guidance.I was provided with her address and landline and mobile phone numbers as follows:

Address: 4th Floor, Department of Health Education and Health Promotion, School of Public Health, Tehran University of Medical Sciences, Poursina St., Ghods St., Islamic Revolution St., Tehran Phone number: 02188989128- Mobile: 09386545847

Mehrpishegan.com is the website address for more information.

13.I know that the Research Ethics Committee can access my information to monitor my rights.

14.I know that if I have any problems or objections to those involved in the research process, I can contact the Research Ethics Committee of Tehran University of Medical Sciences at:

Sixth floor, Ghods St, Keshavarz Boulevard, Secretariat of the Institutional Research Ethics Committee, School of Public Health & Allied Medical Sciences, Tehran University of Medical Sciences,
Phone number: 81633626 -81633613

I have read and understood the above and based on that, I express my informed consent to participate in this research.
Participant's signature

I, Fatemeh Rahimi, consider myself obliged to fulfill the obligations related to the executor in the above provisions and I undertake to try to ensure the rights and safety of the participant in this research. Principal Researcher:

Fatemeh Rahimi signature
